# Comparative immunoexpression of ICAM-1, TGF-β1 and ki-67 in periapical and residual cysts

**DOI:** 10.4317/medoral.21346

**Published:** 2016-12-06

**Authors:** Rafaela Martins, Luciana Armada, Teresa-Cristina dos Santos, Fabio-Ramoa Pires

**Affiliations:** 1 DDS, MSc, Post Graduation. Program in Dentistry, Estácio de Sá University; 2 DDS, PhD, Professor. Post Graduation Program in Dentistry, Estácio de Sá University; 3 DDS, MSc, Professor. Oral Pathology, School of Dentistry, State University of Rio de Janeiro; 4 DDS, PhD, Professor. Post Graduation Program in Dentistry, Estácio de Sá University

## Abstract

**Background:**

This study compared the immunohistochemical expression of ki-67, transforming growth factor beta 1 (TGF-β1) and intercellular adhesion molecule-1 (ICAM-1) in inflammatory periapical cysts and residual cysts.

**Material and Methods:**

The study sample was composed by 25 periapical cysts and 25 residual cysts and immunohistochemical reactions were carried out using antibodies directed against ICAM-1, TGF-β1 and ki-67. Clinical, radiological, gross, histological and immunohistochemical data were tabulated for descriptive and comparative analysis using the SPSS software and differences were considered statistically significant when *p*<0.05%.

**Results:**

There were no differences between the expression of ICAM-1 (*p*=0.239) and TGF-β1 (*p*=0.258) when comparing both groups. Ki-67 labeling index was higher in residual cysts compared to periapical cysts (*p*=0.017).

**Conclusions:**

Results from the present study suggest that some specific inflammatory stimuli on residual cysts would modulate their mechanisms of etiopathogenesis, growing and repair.

**Key words:**Periapical cyst, radicular cyst, residual cyst, transforming growth factor beta 1 (TGF-β1), intercellular adhesion molecule 1 (ICAM-1), ki-67.

## Introduction

Chronic inflammatory periapical lesions (periapical granulomas and periapical cysts) arise from inflammatory periapical stimuli associated with necrosis of the pulp. Periapical granulomas are composed by a fibrous connective tissue infiltrated by chronic inflammatory cells sometimes presenting isolated epithelial islands. Periapical cysts (PC) are characterized by the presence of a cavity lined by non-keratinizing stratified squamous epithelium, containing an inflammed and hemorrhagic fluid, apart from a similar inflammed connective tissue than the one observed in periapical granulomas (1-3). The most accepted theory for PC pathogenesis consider that the inflammatory infiltrate on the periapical region estimulates the proliferation of the odontogenic epithelial remnants (epithelial rests of Malassez) that exist on periapical granuloma and that this epithelial cells will generate the cystic epithelium lining (4).

When the tooth associated with a PC is extracted but the cyst is inadvertently not removed in the same surgical procedure, the remaining lesion is called residual cyst (RC), an entity diagnosed by the association of clinical, radiological and histological features (5,6). The exact pathogenesis of RC and the proliferative capacity of its epithelium are not well-established and it is still a matter of debate if these cysts continue to grow after removal of the associated tooth, if they stop growing or if they can show regression. As in most cases where RC is a clinical-radiological diagnostic hypotheses treatment is based on conservative surgical removal of the cyst, due to the differential diagnosis with other maxillary odontogenic and non-odontogenic cysts and tumors, few RC are followed-up without surgical management to show their natural biological behavior.

The participation and regulation of specific cytokines and how they correlate with the inflammatory infiltrate in inflammatory periapical diseases is not fully understood (7). Intercellular adhesion molecule 1 (ICAM-1) is important for leukocyte adhesion on endothelial cells and their migration to the inflammatory foci (8,9). Transforming growth factor beta 1 (TGF-β1) is an important cytokine on several biological processes, including tissue repair, angiogenesis and bone metabolism (10,11). Expression of antigens associated with proliferative activity, such as the ki-67 protein, has been associated with the proliferative potential in both odontogenic cysts and tumors (12). These and others antigens have been studied in inflammatory periapical diseases, but their comparative expression on RC has not been previously described. Thus, the aim of the present study was to compare the immunoexpression of ICAM-1, TGF-β1 and ki-67 in a series of PC and RC.

## Material and Methods

The studied sample was composed by 25 PC and 25 RC retrieved from the files of the Oral Pathology laboratories, Estácio de Sá University and State University of Rio de Janeiro, Brazil. Clinical, radiological and gross information from all cases were obtained by revision of the laboratory submission forms from each case. Five-µm hematoxylin and eosin stained histological sections from the 50 paraffin blocks were evaluated under light microscopy and the intensity of the inflammatory infiltrate was classified in focal/mild and moderate/intense according with the number and distribution of inflammatory cells in representative medium power magnification fields (20x). The presence of cholesterol clefts was also evaluated on medium-power fields.

Three-µm sections on silanized slides were used for the immunohistochemical reactions. Sections were deparaffinized, hydrated and submitted to heat-induced antigen retrieval with a pH 6.0 citrate buffer on microwave. Endogenous peroxidase was blocked by using a 3% hydrogen peroxyde solution and sections were incubated overnight with primary antibodies anti-ICAM-1 (clone G-5, Santa Cruz Biotechnology, Santa Cruz, CA, 1:1000), TGF-β1 (clone V, Santa Cruz Biotechnology, Santa Cruz, CA, 1:200) and ki-67 (clone MIB-1, Dako, Carpinteria, CA, 1:100). Sections were then incubated with secondary antibodies conjugated with a sptreptavidin-biotin-peroxidase system (LSAB; Dako, Carpinteria, CA) and diaminobenzidine (DAB; Dako, Glostrup, Denmark) was used as the chromogen, followed by staining with Carazzi´s hematoxylin. Positive (according to manufacturer´s suggestions) and negative (omission of primary antibodies) controls were used in all reactions.

ICAM-1 immunoexpression was evaluated in endothelial cells, epithelial cells and mononuclear inflammatory cells, while TGF-β1 immunoexpression was evaluated in fibroblasts from the fibrous capsule. Expression was considered focal/mild when up to approximately 25% of the studied cells showed poisitive immunoreaction and moderate/intense when more than 25% of the studied cells were immunoreactive. The proliferative index was established after couting the number of epithelial cells showing a positive nuclear staining for ki-67 and the total number of epithelial cells in 4 high-power fileds (40x) under light microscopy. The percent of ki-67 positive cells on the total number of epithelial cells was obtained in the 4 fields and the final value was represented by the mean of the 4 studied fields. The selected areas were chosen by the integrity of the epithelial lining and the most representative positiveness.

All clinical, radiological, gross, histological and immunohistochemical data were tabulated and descriptively and comparatively analysed on both groups (PC and RC) using the Statistical Program for Social Sciences (SPSS, version 20) (chi-square and T test), with a significance level of 5% (*p*<0.05). The study was approved by the Ethics in Research Committee from the Estácio de Sá University under the protocol 650.018. Informed consent was obtained from all subjects involved in the study.

## Results

Mean age of the patients affected by PC (40.5 years, ranging from 9 to 67 years) was lower than mean of age of the RC affected patients (58.4 years, ranging from 31 to 82 years) (*p*≤0.0001). PC affected mostly females (13 cases, 52%) while most RC affected males (16 cases, 64%). PC affected the anterior maxilla (14 cases, 56%), posterior mandible (7 cases, 28%) and posterior maxilla and anterior mandible (2 cases each, 8% each). RC affected the anterior maxilla (12 cases, 48%), posterior mandible (9 cases, 36%), posterior maxilla (3 cases, 12%) and anterior mandible (1 case, 4%). There were no statistically significant differences when comparing PC and RC regarding the presence of symptoms, swelling in the affected area and anatomical distribution of the lesions. Mean volume of the surgical specimens submitted to the laboratories showed that PC presented a mean of 1984.5 mm3 (ranging from 24 to 21000 mm3) and RC a mean of 1882.5 mm3 (ranging from 108 to 12600 mm3) (*p*=0.919). The mean largest radiological diameter of PC was 26.1 mm (ranging from 10 to 50 mm) in comparison to 21 mm for RC (ranging from 10 to 45 mm) (*p*=0.107). The mean interval of time from extraction to diagnosis of the RC was 266 months, ranging from 36 to 504 months. Mean time of complaint reported by the patients affected by PC was 21.3 months (ranging from 4 to 60 months) in comparison with 47.8 months in RC (ranging from 1 to 204 months) (*p*=0.397).

**Figure 1:**
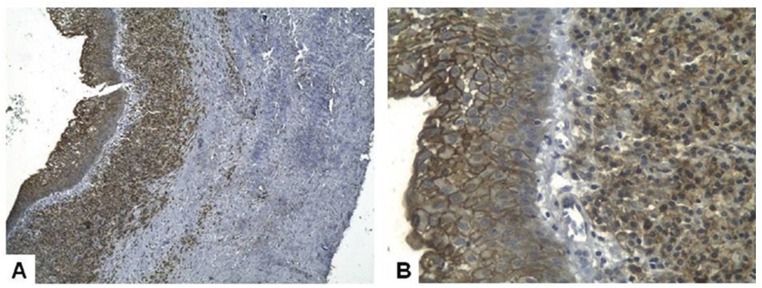
A. ICAM-1 immunoexpression on epithelial and chronic inflammatory cells in a residual cyst (Immunoperoxidase). B. Detail of the epithelial and inflammatory cells expressing ICAM-1 seen in A (Immunoperoxidase, 40x).

Analysis of the histological (intensity of the inflammatory infiltrate and presence of cholesterol clefts) and immunohistochemical (expression of ICAM-1 e TGF-β1) parameters showed no statistically significant differences when comparing PC and RC ( Table 1 and Figs. 1,*p*). Mean proliferative index measured by ki-67 expression on lining epithelial cells was 1.25% for PC (ranging from 0% to 5.31%) in comparison with 3.51% in RC (ranging from 0% to 16.3%) (*p*=0.017) (Fig. 3).

**Table 1:**
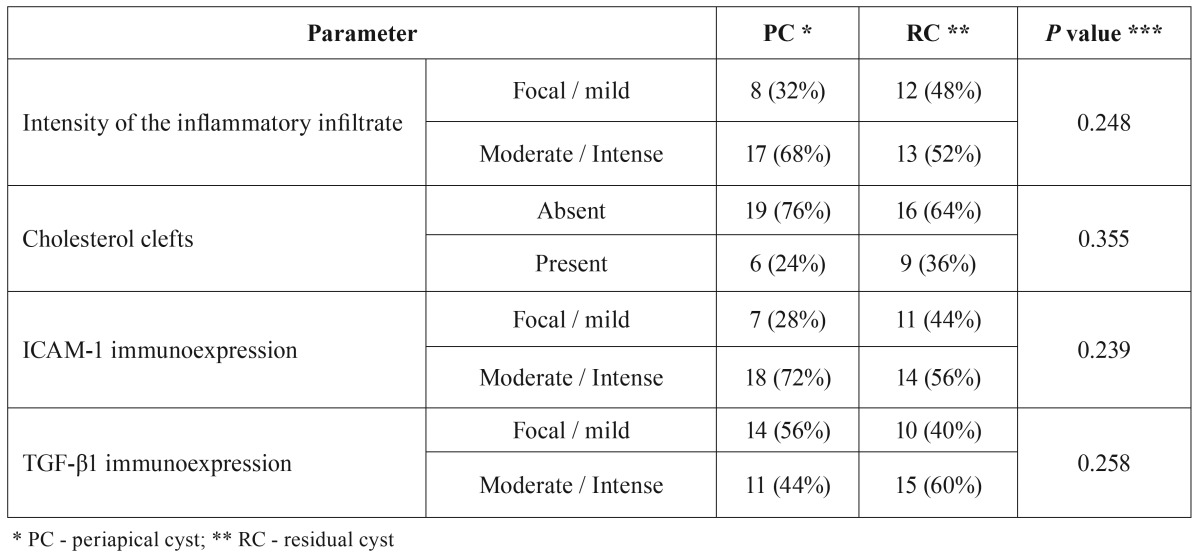
Comparison of histological and immunohistochemical parameters from periapical cysts and residual cysts.

**Figure 2:**
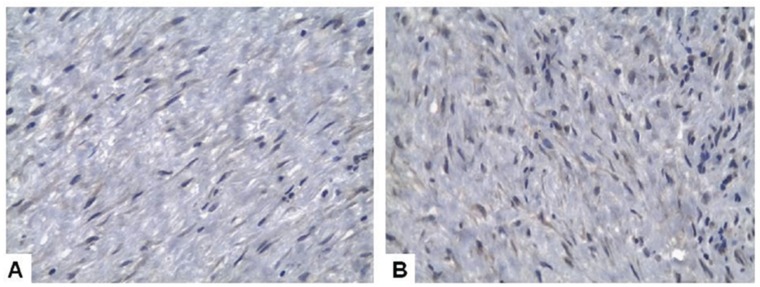
A and B. TGFβ1 immunoexpression in fibroblasts from the fibrous connective tissue capsule in a residual cyst (A and B - Immunoperoxidase, 40x).

**Figure 3:**
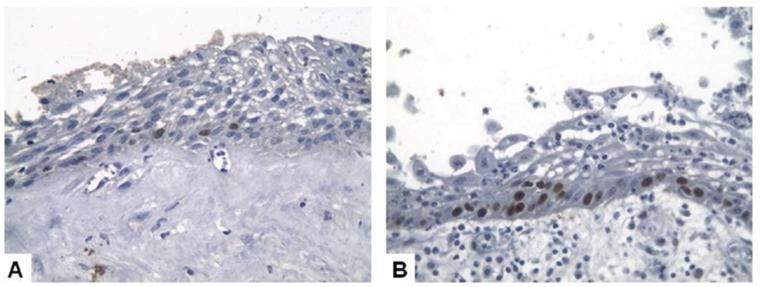
Ki-67 immunoexpression in basal and immediately suprabasal cells from the epithelial lining in a periapical cyst (A) and a residual cyst (B) (A and B - Immunoperoxidase, 40x).

In PC, there were no statistically significant differences when comparing the mean proliferative index by the presence of symptoms (*p*=0.915) and the presence of primary or secondary infection on the associated teeth (*p*=0.364). There was no positive association of the largest radiological diameter of PC with the proliferative index (*p*=0.961). In RC there was also no statistically significant difference when comparing the mean proliferative index by the presence of symptoms (*p*=0.378). There was no positive association of the largest radiological diameter of RC with the proliferative index (*p*=0.790). The mean proliferative index in RC showed that comparing the cases with rupture of the cortical plates (4.94%) and cases with no rupture of the cortical plates (1.54%), the difference was close to statistical significance (*p*=0.054).

## Discussion

RC is considered a persistent PC that was not surgically removed when the associated tooth was extracted. Although this pathogenetic mechanism for RC seems easily understood, there are no convincingly scientific evidences that can confirm this origin. Most RC do not have previous radiological documentation and the few cases where it is possible to retrieve previous image data do not show any conclusive evidence (histological report) that the preceding periapical lesion was a PC. Both cysts are microscopically very similar, but some RC can show only some foci or almost no inflammation, difficulting the confirmation of their inflammatory origin. The present results showed, on the contratry, that PC and RC can be histologically quite similar, with almost no differences on the amount, composition and distribution of the inflammatory infiltrate. One possible explanation would be the short interval of time from tooth extraction and removal of the RC, allowing persistence of the inflammatory component, but the present results showed that the lower interval of time was 36 months (mean of 266 months), offering enough time for reduction of the inflammatory component.

It should be important to consider that other stimuli persist on RC allowing the maintenance of the inflammatory infiltrate and favoring its growth. Physiological resorption of the alveolar bone after tooth extraction can produce a reduction of the bone thickness covering the RC, increasing the possibility that the fibrous capsule of the cyst stays in close proximity to the adjacent soft tissues. This phenomenon is supposed to increase the possibility that other inflammatory stimuli, such as trauma and secondary infection, induce the maintenance of the inflammatory infiltrate on these cysts. Mean expression of ki-67 on RC associated with rupture of the peripheral cortical plates was higher that in RC showing integrity of the cortical plates. However, the number of cases involved in the study and the absence of computed tomographic analysis of all cortical plates from all lesions limited the interpretation of these data. The present results have additionally demonstrated that for both PC and RC the mean largest radiological diameter of the lesions did not positively correlate with the proliferative index, reinforcing that increasing on the epithelial proliferative capacity is not simply associated with their natural expected growth. We have also shown that the presence of symptoms, suggesting association with secondary infection, reinfection and/or chronic trauma, was not associated with a higher epithelial proliferative index. Perhaps another unknown factor, possibly associated with an intrinsic proliferative capacity of the epithelial lining cells, can be responsible for these differences on proliferative potential.

Mean age of patients affected by RC was, as expected and previously demonstrated (13-17), higher than the mean age of patients with PC. It is expected that RC can stay asymtomatically growing on the maxillary bones for years after tooth extraction, and diagnosis is usually established late due to secondary infection or when diagnosed in incidental radiological analysis.

ICAM-1 expression on endothelial cells plays an important role on the migration of activated leukocytes to the site of inflammation and is also important on the adaptative immunity (9). ICAM-1 vascular expression on healthy pulp tissue is practically absent, in contrast with the increased expression found in inflammed pulps (18). Activated endothelial cells, epithelial cells and mononuclear inflammatory cells found in PC express ICAM-1, reinforcing the importance of this adhesion molecule on the inflammatory mechanisms that modulate it (8). The present results showed that, when comparing PC and RC showing similar inflammatory infiltrate intensity, ICAM-1 expression was similar, suggesting that its expression seems to be associated with the presence and intensity of inflammation more than other biological characteristics of the cysts. Although moderate/intense ICAM-1 expression in PC was more common than in RC, the difference was not statistically significant.

TGF-β1 regulates several biological processes such as proliferation, survival, differentiation, apoptosis and cellular migration, and extracellular matrix production, showing an important role on immunological reactions, angiogenesis, tissue repair and bone metabolism (10,11). TGF-β1 expression in PC and in periapical granulomas seems to be associated with its role on the repair of the periapical destruction secondary to their growth (19). TGF-β1 RNAm expression was significantly higher in stable/inactive compared with active/progressive granulomas, suggesting that expression of this gene can be used as a marker for tissue repair and predictability of the biological behavior of these lesions (20). In contrast, the presence of TGF-β1 has been demonstrated in PC and periapical granulomas, but not in scar tissue, and showed positive correlation with the size of the lesions (21). Piattelli *et al.* (22) observed a higher TGF-β1 expression in odontogenic keratocysts than in PC and dentigerous cysts and associated this finding to the higher growth potential of keratocysts. Other studies have reported a similar TGF-β1 expression in PC and periapical granulomas (3,19,23) and Teixeira-Salum *et al.* (24), evaluating the levels of several cytokines in PC and periapical granulomas, observed that granulomas expressed higher levels of TGF-β. These scientific evidences have suggested that TGF-β1 is an important factor in periapical repair, through inhibition of bone resorption and promotion of bone remodelling and soft tissue repair. It has been demonstrated that TGF-β1 stimulates collagen synthesis, neovascularization and proliferation of fibroblasts (19). Andrade *et al.* (25), however, showed a possible role for TGF-β1 on stabilization of periradicular disease through immunosupression and its regulatory effects on inflammation, observing a more evident immunoexpression on more inflammed lesions. The present results did not show statistically significant differences on immunoexpression of TGFβ-1 when comparing PC and RC, possibly due to the presence of a similar inflammatory component on the studied cysts from both groups.

The expression of antigens associated with cellular proliferation has been associated with the growth properties of several odontogenic cysts and tumors. The ki-67 antigen is a nuclear protein expressed in proliferating cells and absent in quiescent cells (26). There are evidences that the expression of ki-67 on the epithelium lining of odontogenic cysts, including PC, can be elevated in the presence of a more intense inflammatory infiltrate (27), suggesting that an increasing in the proliferative activity on these cysts could be directly influenced by inflammation. Additionally, ki-67 expression seems to be elevated in hyperplastic epithelium in contrast with atrophic epithelium in PC (12,28). The cysts included in both groups evaluated in our study showed very similar histological features and intensity of inflammatory infiltrate, features reflected by the similar ICAM-1 and TGF-β1 immunoexpression. The present results suggest that the intensity of the inflammatory infiltrate can only partially regulate the ki-67 epithelial immunoexpression as, when we compared PC and RC showing similar inflammatory component, ki-67 mean expression in the latter was higher than in the former. These features support the hypothesis that other stimuli, apart from the inflammation derived from the pulp and periapical tissues, such as proximity of the cyst from the oral soft tissues due to rupture of the cortical plates or secondary infection, can modulate the proliferative capacity of the RC epithelium.

In conclusion, the present results did not show statitically significant differences on the immunoexpression of ICAM-1 and TGF-β1 when comparing PC and RC and showed that ki-67 epithelial immunoexpression was higher in RC than in PC. Other studies should be encouraged for a better comprehension of the mechanisms associated with the epitheilal proliferation in RC, helping understanding its pathogenesis and defining adequate management strategies.
